# Challenges and opportunities for pharmaceutical pricing and reimbursement policies

**DOI:** 10.1186/2052-3211-8-S1-E1

**Published:** 2015-10-05

**Authors:** Sabine Vogler, Nina Zimmermann, Alessandra Ferrario, Veronika J Wirtz, Zaheer-Ud-Din Babar

**Affiliations:** 1 WHO Collaborating Centre for Pharmaceutical Pricing and Reimbursement Policies, Health Economics Department, Gesundheit Österreich GmbH (Austrian Public Health Institute), Vienna, 1010, Austria; 2 LSE Health and Department of Social Policy, London School of Economics and Political Science, London, WC2A 2AE, UK; 3 Department of Global Health, Boston University School of Public Health, Boston, MA 02118, USA; 4 School of Pharmacy, Faculty of Medical and Health Sciences, University of Auckland, Private Mail Bag 92019, Auckland, New Zealand

## 

Even though access to affordable medicines is a human right, it is not ensured world-wide. The Priority Medicines Report 2013 [1] identified pharmaceutical gaps that continue to remain: diseases of public health importance for which pharmaceutical treatments either do not exist or are inadequate (see also K4). Where adequate treatments were available, access might be limited due to high costs of the medicines that can neither be funded by individuals nor by the communities.

Ensuring equitable access to safe and effective medicines is a complex task. To prevent individuals from incurring into financial hardship when accessing health care, including medicines and to reduce the barriers to medicines access, quality of care and increasing equity, the World Health Organization (WHO) has been promoting Universal Health Coverage (UHC). During the last years a number of countries worldwide have been working towards UHC.

Still, there is a disproportion in resource allocation for health care, including medicines, between countries at different levels of income. While expenditure is not necessarily a good indicator of better access, it is worth noting that in 2005/2006 (latest data available at international level) for example, 16% of the world's population living in high-income countries accounted for over 78% of global expenditures on medicines [2].

## Equitable access to new high-priced medicines

Increasingly, funding of medicines has also become a challenge in high-income countries. Ageing populations and increasing prevalence of non-communicable diseases play an important role in this in addition to two main, more recent factors which are responsible for continuing pressure on public budgets. First, the global financial crisis hit hard some of the more affluent countries such as European countries (see E2, one strand of the PPRI Conference being devoted to the crisis). Second, a number of new high-priced medicines, including medicines for which no treatment was previously available, have been marketed, and more are in the pipeline. While this is promising for patients, it has been met with concern by policy-makers and payers since these medicines tend to be sold at premium prices. Frequently, these are medicines in the areas of oncology and/or medicines for rare diseases (orphan medicines). For the latter, special policies were designed in order to incentivize the pharmaceutical industry to do research in this field of presuming low volumes [3]. However, several orphan medicines rather have high sales volumes, and in total, orphan diseases are not so rare [4]. The best-known example for a high-priced medicine, however, is for the treatment of hepatitis C: in 2014, sofosbuvir challenged the publicly funded health care systems of numerous countries the world over and triggered discussions about the appropriateness of existing policy options to deal with high-priced medicines.

## Pharmaceutical pricing and beyond

In order to confront challenges of access to new, potentially high-priced medicines, there is a need for the effective use of existing policies and for new and innovative policies that are not solely limited to pricing.

In March 2015, the WHO Regional Office for Europe published a report about access to new medicines [5] that offers a review of interventions that policy-makers might choose to manage the market entry of high-priced medicines in order to improve patient access to potentially innovative medicines and to reward and incentivize industry for research while ensuring financial sustainability (see also K2). The report made clear that in addition to activities alongside the market entry of medicines, policy-makers should consider undertaking measures before launch, such as horizon scanning and planning far in advance, and post-launch activities to strengthen the compliance to guidelines and formularies and to improve the medicine management at the interface of in-patient and out-patient sectors (see also Strand 3 of the PPRI Conference, E3).

Further, more collaborative approaches between the different actors have been suggested, including a closer cooperation between the regulatory authorities and public bodies for pricing and reimbursement. In order to reduce possible overlaps between the licensing and the pricing and reimbursement processes and ensure early access to promising new medicines, the European Medicines Agency launched in March 2014 the pilot project of ‘adaptive pathways’ which foresees an early approval of a medicine for a restricted patient population based on small initial clinical studies. The first approval is followed by progressive adaptations of the marketing authorisation to expand access to the medicine to broader patient populations based on ‘real-life’ clinical data on utilization [6]. In addition to a stronger cooperation between authorities, increased dialogue with other stakeholders has been recommended, in particular discussions on what constitutes a fair reward for industry innovation while still preserving access for patients [5]. Patients and citizens must not be forgotten in the debate. It was recommended exploring ways of how to better involve them since their potential to strengthen the quality and legitimacy of the decision-making process has been acknowledged [1].

## New challenges and opportunities

In the field of pricing, common policies are increasingly being questioned because they appear to be no longer able to deal with new challenges such as high-priced medicines. In the Council conclusions on ‘Innovation for the benefit of patients’ as of 6 December 2014 European policy-makers noted with concern that, due to the very high prices of some innovative medicines, patients do not always have access to innovative treatments [7]. Concerns have been voiced that medicine shortages that have increasingly been observed also in higher-income countries, are, among other factors, attributable to existing pricing policies [8]. External price referencing (i.e. international price comparison) is the commonly applied pricing policy in European countries [9] and, increasingly, in several countries over the world [10]. This policy tends to incentivize marketing authorization holders to first launch medicines in countries with higher price levels, and delay, and even refrain from, launching in low-price countries [11]. While this has been long known, EPR's possibly limiting impact access has been recently observed particularly in countries that were hit hard by the crisis and related to new high-priced medicines – the two main recent challenges mentioned above.

As a result, alternative pricing and funding models have been implemented or are under discussion. In recent years, managed-entry agreements were introduced in several countries. However, while they are instruments to manage uncertainty and to allow faster patient access to new medicines, with possibly limited data on their effectiveness, they tend to contribute to intransparency due to their confidential contents [12,13]. Although value-based pricing as an integrative pricing and reimbursement policy is only in place in few countries (e.g. Sweden), tools (e.g. health technology assessments) aiming to assess value are applied in several countries [14]. Discussions have started whether, and how, the economic situation of a country could be considered into pricing policies [15].

At the same time, new opportunities could be seized. The recent and future patent expiries of high-cost, frequently biotechnological, medicines is very likely to allow patient access to highly effective medicines at lower prices and to offer potential savings to public payers. However, it has not been fully explored yet how to make best use of biosimilar medicines, and even generics.

The 2015 Vienna PPRI Conference provides a forum to discuss these issues with leading experts including Suzanne Hill (WHO, K1) and Andy Gray (University of KwaZulu-Natal, K3) and different stakeholders. Strand 1 of the PPRI Conference is particularly dedicated to current and recurrent challenges in pharmaceutical pricing and reimbursement and possible opportunities, with key-notes of Veronika Wirtz (Boston University, K4) and Arnold Vulto (Erasmus University Hospital) as well as presentations about policies to deal with high-cost medicines (e.g., O1, O2, O9, O14) and experiences with generic policies (O6).

## References

[B1] Kaplan WA Wirtz VJ Mantel-Teeuwisse AK Stolk P Duthey B Laing R Priority Medicines for Europe and the World. 2013 Update 2013 World Health Organization

[B2] Lu Y Hernandez P Abegunde D Edejer T The world medicines situation 2011. Medicine expenditures 2011 World Health Organization, Geneva

[B3] Kanavos P Nicod E What is wrong with orphan drug policies? Suggestions for ways forward Value in Health 2012 15 8 1182 1184 10.1016/j.jval.2012.08.2202 23244822

[B4] Graf von der Schulenburg JM Frank M Rare is frequent and frequent is costly: rare diseases as a challenge for health care systems The European Journal of Health Economics 2014 16 2 1 6 10.1007/s10198-014-0639-8 25355295

[B5] WHO Regional Office for Europe Access to new medicines in Europe: technical review of policy initiatives and opportunities for collaboration and research 2015 Copenhagen

[B6] European Medicines Agency (EMA) Pilot project on adaptive licensing London 19 March 2014. http://www.ema.europa.eu/docs/en_GB/document_library/Other/2014/03/WC500163409.pdf

[B7] European Commission Council conclusions on innovation for the benefit of patients (2014/C 438/06) 2014 Brussels

[B8] De Weerdt E Simoens S Hombroeckx L Casteels M Huys I Causes of drug shortages in the legal pharmaceutical framework Regulatory Toxicology and Pharmacology 2015 71 2 251 258 10.1016/j.yrtph.2015.01.005 25591547

[B9] Leopold C Vogler S Mantel-Teeuwisse AK de Joncheere K Leufkens HG Laing R Differences in external price referencing in Europe-A descriptive overview Health Policy 2012 104 1 50 60 10.1016/j.healthpol.2011.09.008 22014843

[B10] Espin J Rovira J de Labry AO Working paper 1: External price referencing – review series on pharmaceutical pricing policies and interventions 2011 Geneva: World Health Organization and Health Action International

[B11] Bouvy J Vogler S World Health Organization Background Paper 8.3 Pricing and Reimbursement Policies: Impacts on Innovation Priority Medicines for Europe and the World “A Public Health Approach to Innovation” Update on 2004 Background Paper 2013 Geneva

[B12] Ferrario A Kanavos P Dealing with uncertainty and high prices of new medicines: A comparative analysis of the use of managed entry agreements in Belgium, England, the Netherlands and Sweden Social Science & Medicine 2015 124 1 39 47 25461860 10.1016/j.socscimed.2014.11.003

[B13] Robertson J Walkom EJ Henry DA Transparency in pricing arrangements for medicines listed on the Australian Pharmaceutical Benefits Scheme Australian health review 2009 33 2 192 199 10.1071/AH090192 19563308

[B14] Paris V Belloni A Value in Pharmaceutical Pricing. OECD Health Working Papers, No. 63 2013 Paris: OECD Publishing

[B15] Rovira J Can EU citizens afford their medicines? The financial crisis and access to medicines in Europe. Presentation at the High Level Meeting of the European Parliament, 16 May 2013

